# Safety and efficacy of CAR-T cell targeting BCMA in patients with multiple myeloma coinfected with chronic hepatitis B virus

**DOI:** 10.1136/jitc-2020-000927

**Published:** 2020-08-13

**Authors:** Lu Han, Jian Zhou, Keshu Zhou, Xinghu Zhu, Lingdi Zhao, Baijun Fang, Qingsong Yin, Xudong Wei, Hu Zhou, Linlin Li, Bengling Xu, Jishuai Zhang, Yongping Song, Quanli Gao

**Affiliations:** 1 Department of Immunology, Affiliated Cancer Hospital of Zhengzhou University and Henan Cancer Hospital, Zhengzhou, China; 2 Department of Hematology, Affiliated Cancer Hospital of Zhengzhou University and Henan Cancer Hospital, Zhengzhou, China; 3 The Shenzhen Pregene Biopharma Company, Ltd, Shenzhen, China

**Keywords:** immunotherapy, immunotherapy, adoptive, clinical trials as topic

## Abstract

**Background:**

Reactivation of hepatitis B virus (HBV) infection is a well-recognized complication in patients with chronic or resolved HBV infection undergoing anticancer therapy. There is a risk of HBV reactivation after infusion of chimeric antigen receptor (CAR) T cells for patients with refractory/relapsed (R/R) multiple myeloma (MM).

**Methods:**

We administered B cell maturation antigen (BCMA) CAR-T cell by infusion to nine patients with R/R MM with chronic or resolved HBV infection. Patient serum was analyzed to determine the expression of five components of HBV and the copy number of HBV DNA. HBV reactivation was defined if a patient re-exhibited hepatitis B surface antigen (HBsAg) or HBV DNA regrowth after CAR-T therapy.

**Results:**

In one patient who was HBsAg-positive, no HBV reactivation was observed during the follow-up period of 9.8 months after administration of anti-HBV drugs before and after CAR-T therapy. Among eight patients with MM who had resolved HBV infection, two patients administered prophylactic anti-HBV drugs did not exhibit HBV reactivation. Of the six patients who did not use prophylactic antiviral drugs, five did not exhibit HBV reactivation, while one showed recurrence of HBsAg without detection of HBV DNA or damage to liver function. The best objective response rate was 100%, and the progression-free survival (PFS) at 12 months was of 88.89% (median PFS was not observed).

**Conclusions:**

These findings showed that BCMA CAR-T cell therapy could be used in patients with R/R MM with chronic or resolved HBV infection and that antiviral drugs should be administered in these patients during CAR-T cell therapy.

## Background

Hepatitis B virus (HBV) infection is a serious health concern. Estimates have shown that more than 257 million people worldwide are infected with HBV and approximately 887,000 people die of HBV infection each year.^1^ HBV reactivation is a well-recognized complication of anticancer therapy and can result in multiple clinical manifestations, ranging from asymptomatic hepatitis to fulminant hepatitis.^2 3^

HBV reactivation occurs in two separate clinical scenarios. First, patients with cancer who were hepatitis B surface antigen (HBsAg) positive (ie, patients with cancer with chronic HBV infection) can experience HBV reactivation, as demonstrated by an increase in serum HBV DNA levels and biochemical or clinical evidence of hepatitis. Second, patients who have a resolved HBV infection (HBsAg-negative with positive anti-hepatitis B core antibody (HBcAb), with or without an antibody to HBsAg (HBsAb)) can experience HBV reactivation.^4–7^ In patients with resolved HBV infection, a low level of HBV replication can be detected for many years in peripheral blood mononuclear cells and the liver,^8 9^ and HBV reactivation can occur during cytotoxic chemotherapy (CT), monoclonal antibody treatment, or bone marrow transplantation, with HBsAg appearance (HBsAg seroreversion).^10–13^

Chimeric antigen receptor (CAR)-T cell therapy is a novel treatment with definite curative effects for some hematological cancers.^14–16^ B cell maturation antigen (BCMA)-based CAR-T cells have significantly improved outcomes in patients with refractory/relapsed (R/R) multiple myeloma (MM), with objective response rates (ORRs) of more than 80% and complete remission (CR) rates of up to 60%.^17–20^ To avoid viral reactivation or fulminant hepatitis, clinical trials of CAR-T cell therapy have generally excluded patients with aggressive MM coinfected with HBV. Thus, to date, the safety and efficacy of CAR-T cell therapy among patients with MM and HBV infection remain unexplored, and data on HBV reactivation in these patients are limited. Therefore, in this retrospective study, we evaluated the safety and efficacy of BCMA CAR-T cell therapy in patients with R/R MM and concomitant HBV infection.

## Methods

### Patient population

This study was conducted at the Affiliated Cancer Hospital of Zhengzhou University and was registered with clinicaltrials.gov (registration number: NCT03664661). The study protocol was approved by the Ethics Committee of the Affiliated Cancer Hospital of Zhengzhou University. Informed consent was obtained from all patients.

Eligibility criteria were as follows: age, 18–70 years; Eastern Cooperative Oncology Group performance score, ≤2 (on a scale of 0–5, with higher scores indicating greater disability); measurable disease, based on serum monoclonal protein (M protein) ≥1.0 g/dL in serum or ≥200 mg/24 hours in urine, serum-free light chain (FLC) concentration of ≥10 mg/dL with an abnormal ratio, or bone marrow plasma cells ≥10%; at least three previous lines of therapy, with each line of treatment having at least one complete treatment cycle; received an immunomodulatory drug (IMiD) and a proteasome inhibitor (PI), or had disease that was refractory to both drug classes; and adequate organ function. One additional criterion included BCMA expression in marrow plasma cells based on flow cytometry analysis. More details on the inclusion and exclusion criteria are given in the online supplementary appendix.

10.1136/jitc-2020-000927.supp1 Supplementary data

HBsAg, HBsAb, hepatitis B e antigen, hepatitis B e antibody (HBeAb), and HBcAb were detected by chemiluminescence by clinical laboratory analyzers using commercially available kits (Roche, Basel, Switzerland) before CAR-T cell therapy. HBV DNA was quantitated by PCR. Patients with active HBV infection prior to CAR-T cell therapy were excluded from the study. Patients who were HBsAg positive were given antiviral drugs during the entire treatment period, and prophylaxis with entecavir (1 mg) or lamivudine (100 mg) was recommended to patients who had resolved HBV infection until the immunoglobulin (Ig) levels recovered. As an indicator of HBV reactivation, hepatitis B serology and HBV DNA levels were closely monitored for all patients when evaluating efficacy during follow-up, and increasing testing number if there were obvious abnormal clinical hepatitis symptoms or the changes of liver function (bilirubin, transaminase, and albumin) after CAR-T cells infusion. HBV reactivation was indicated by (1) HBsAb absence and HBsAg appearance in patients who were HBsAg negative, with or without HBsAb; and (2) increase in HBV DNA levels by at least 10-fold or 1×10^9^ copies/mL.^21^ Antiviral treatment was initiated as soon as reactivation was detected. None of the patients had previously received hepatitis B vaccines.

### Preparation and administration of CAR-T cells

**Figure 1:**
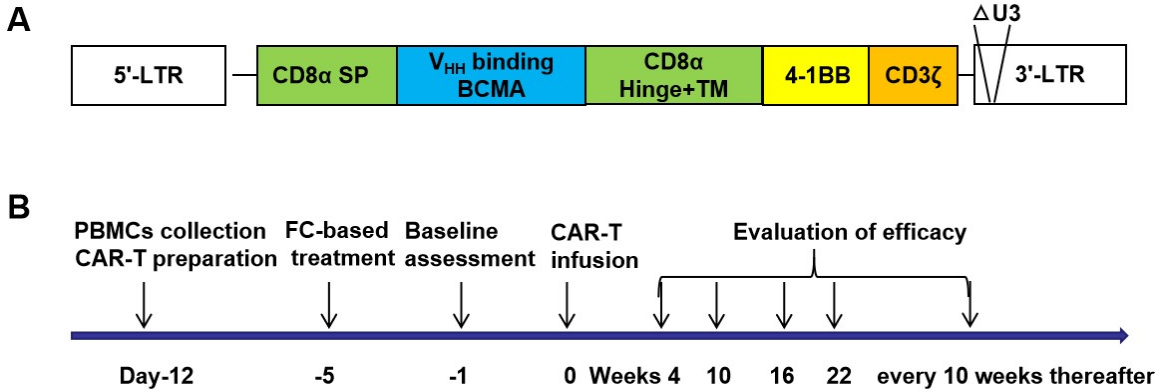
BCMA CAR-T cells and clinical treatment protocols. (A) Diagram of BCMA CAR, composed of a human CD8α signal peptide (CD8α sp), BCMA binding domain (V_HH_, variable domain of the heavy chain antibody), human CD8α hinge and transmembrane domain (CD8α hinge +TM), human 4-1BB cytoplasmic domain, and human CD3ζ cytoplasmic domain (CD3ζ). (B) Clinical treatment protocol. Patients underwent lymphocyte isolation to obtain peripheral blood lymphocytes on day –12, and cells were transduced, cultured, and expanded. The first day of CAR-T infusion was established as study day 0, and patients underwent fludarabine/cyclophosphamide-based lymphodepletion chemotherapy on day –5. On day –1; weeks 4, 10, 16, and 22; and every 10 weeks thereafter, efficacy was evaluated. △U3, U3 region deletion; BCMA, B cell maturation antigen; CAR, chimeric antigen receptor; LTR, long terminal repeat; sp, signal peptide.

BCMA CAR-T cells were manufactured according to Good Manufacturing Practices in the immunology laboratory of the Affiliated Cancer Hospital of Zhengzhou University. Cells expressing anti-BCMA CAR (figure 1A) were expanded over a period of 12 days, and CAR-T cells on day 12 were composed of a variable proportion of CD4^+^ and CD8^+^ T cells by Fluorescence Activating Cell Sorter (FACS) analysis (online supplementary table 1). Patients underwent lymphodepletion with fludarabine and cyclophosphamide, followed by infusion of CAR-T cells on day 0. On day –1; weeks 4, 10, 16, and 22; and every 10 weeks thereafter, efficacy was evaluated (figure 1B). CAR DNA copy numbers were determined to evaluate CAR-T cells expansion and persistence. Additional information on this approach is provided in the online supplementary appendix.

### Evaluation of safety

Adverse events were graded according to the National Cancer Institute’s Common Terminology Criteria for Adverse Events (NCI CTCAE V.4.03), except for cytokine-release syndrome (CRS). CRS was defined and graded according to published criteria proposed by Lee *et al*.^22^ Several serum biomarkers, including C-reactive protein (CRP), ferritin, and cytokines, were used to evaluate adverse events. Cytokines indicative of a response to the CAR-T cells were determined by ELISA kits (additional information is provided in the online supplementary appendix).

### Evaluation of clinical response

The efficacy of the CAR-T cell therapy was evaluated according to the response criteria in the International Myeloma Working Group consensus recommendations.^23^ Patients were closely followed by bone marrow cytology or biopsy examination for plasma cells, minimal residual disease (MRD) of the bone marrow by flow cytometry, serum protein electrophoresis, immunofixation electrophoresis of serum and urine, serum FLC, CT scanning, and MRI.

### Statistical analysis

The data were analyzed with GraphPad Prism V.6.0 and SPSS Statistics V.17.0. Data were presented as the mean±SD. Missing data were not imputed unless otherwise specified. Progression-free survival (PFS) and associated 95% CIs were calculated using the Kaplan-Meier method.

## Results

### Clinical and hematological data for the patient cohort

Between July 11, 2018, and April 17, 2019, nine patients with R/R MM were enrolled in this study. The clinical and hematological characteristics are summarized in table 1. According to the M-protein determination, the Ig or light-chain (LC) subtypes were IgG λ in three cases; IgG κ in two cases; IgA κ in one case; IgA λ in one case; and κ LC in two cases. Five out of nine patients had received at least three lines of prior therapy, including CT, IMiD, and PI, whereas four had received CT with either IMiD or PI. In addition, two patients had also received autologous hematopoietic stem cell transplantation. Anemia, bone lesions, an abnormal FLC ratio, a high level of β2-microglobulin, and elevated lactate dehydrogenase were commonly observed in most patients at the time of the enrollment. BCMA-positive plasmablasts were observed in all cases (3.0%–81.8%). Four patients had existing extramedullary lesions.

**Table 1:** Clinical and hematological data and dosage of CAR-T cells in nine patients with R/R MM

Patient*	Sex/age	Subtype	Lesion	Lines of prior therapy	Auto- HSCT	PI†	IMiD‡	Clonal BM plasma cells, %	BCMA of plasma cells, %	Serum M protein, g/L	β_2_-MG, mg/L	LDH, iu/L	HB, g/L	Bone lesion§	FLC ratio	CAR^+^ T infused ×10^6^/kg
1	M/48	IgG, κ	BM	4	No	Bortezomib	No	38.2	64.4	58.1	2.4	151	91	Yes	13.600	5
2	F/63	IgG, λ	BM, EM**¶**	3	No	Bortezomib	Thalidomid	1.2	24.5	3.6	2.5	135	97	Yes	0.485	5
3	F/43	IgG, κ	BM	8	Yes	No	Thalidomid	0.2	3.0	1.8	2.2	162	122	Yes	0.756	5
4	M/55	κ	EM**¶**	3	No	Bortezomib	Lenalidomide/ Thalidomid	1.2	3.6	0**	1.9	172	124	Yes	74.671	5
5	M/51	κ	BM, EM**¶**	3	No	Bortezomib	Thalidomid	4.0	81.8	4.6	4.3	161	123	Yes	617.048	5
6	M/59	IgG, λ	EM**¶**	6	No	Bortezomib	No	0.6	5.8	2.6	2.6	196	123	Yes	<0.004	5
7	F/51	IgG, λ	BM	3	No	Bortezomib	No	39.0	5.7	3.5	3.5	320	103	Yes	1.040	5
8	F/59	IgA, λ	BM	6	Yes	Bortezomib	Lenalidomide	22.2	74.3	3.9	3.2	150	101	Yes	0.069	5
9	F/47	IgA, κ	BM	3	No	Bortezomib	Thalidomid	32.2	61.3	8.3	1.6	173	120	Yes	1.344	5

*Patients are listed sequentially in the order which they were treated.

†PIs: Bortezomib.

‡IMiDs (Lenalidomide/Thalidomid).

§Bone lesions: one or more osteolytic lesions on ECT, CT, or PET-CT.

¶Four patients exhibited EM lesions. Patient 2 and patient 6 had tumor infiltration in chest; patient 4 plasmacytoma on pars lumbalis; patient 5 had tumor infiltration in the pleura.

**κ monoclonal immunoglobulin appeared in urine.

Auto-HSCT, autologous hematopoietic stem cell transplantation; BCMA, B cell maturation antigen; BM, bone marrow; CAR, chimeric antigen receptor; CT, computed tomography; ECT, emission computed tomography; EM, extramedullary; FLC, free light chain; HB, hemoglobin; IMiDs, immunomodulatory drug; LDH, lactate dehydrogenase; β2-MG, β2-microglobulin; MM, multiple myeloma; PET-CT, positron emission tomography -computed tomography; PIs, proteasome inhibitors; R/R, relapsed/refractory.

Among the nine patients, all had HBV infection or previous HBV infection (table 2). Specifically, we detected HBsAg positive, HBeAb positive, and HBcAb positive in one case; HBeAb positive and HBcAb positive in one case; HBcAb positive in one case; HBsAb positive, HBeAb positive, and HBcAb positive in two cases; and HBsAb positive and HBcAb positive in four cases. The HBV DNA level for all patients was below the detection limit (<100 IU/mL).

**Table 2:** Baseline characteristics and outcomes

Patient	HBsAg	HBsAb	HBeAg	HBeAb	HBcAb	HBV DNA, IU/mL	ALT, U/L	AST, U/L	Bilirubin total µM	Albumin, G/L	Previous heap hepatitis	Antivira therapy	HBsAg*	HBsAb*	HBeAg*	HBeAb*	HBcAb*	HBV DNA, IU/mL*
1	Negative	Negative	Negative	Positive	Positive	<100	14	23	9.6	33.9	Yes	No	Negative	Positive	Negative	Negative	Positive	<100
2	Negative	Positive	Negative	Negative	Positive	<100	7	13	7.5	34.2	Yes	Entecavir	Negative	Negative	Negative	Negative	Negative	NA
3	Negative	Negative	Negative	Negative	Positive	<100	14	8	15.9	49.6	Yes	Lamivudine	Negative	Positive	Negative	Negative	Positive	<100
4	Negative	Positive	Negative	Positive	Positive	<100	11	12	9.1	42.6	Yes	No	Negative	Positive	Negative	Negative	Negative	<100
5	Negative	Positive	Negative	Negative	Positive	NA	15	18	14.7	40.2	Yes	No	Negative	Positive	Negative	Negative	Negative	NA
6	Positive	Negative	Negative	Positive	Positive	<100	21	32	11.5	38.9	Yes	Entecavir/ Lamivudine	Positive	Negative	Negative	Positive	Positive	<100
7	Negative	Positive	Negative	Positive	Positive	NA	37	20	11.3	36.2	Yes	No	Negative	Positive	Negative	Negative	Negative	<100
8	Negative	Positive	Negative	Negative	Positive	NA	9	15	4.8	36.8	Yes	No	Negative	Positive	Negative	Negative	Positive	NA
9	Negative	Positive	Negative	Negative	Positive	<100	10	15	7.3	41.8	Yes	No	Positive	Negative	Negative	Negative	Positive	<100

*Immunological detection of HBV in serum after BCMA CAR-T infusion in 6 to 12 months.

ALT, alanine transferase; AST, aspartate transaminase; BCMA, B cell maturation antigen; CAR, chimeric antigen receptor; HBcAb, hepatitis B core antibody; HBeAb, hepatitis B e antibody; HBeAg, hepatitis B e antigen; HBsAb, hepatitis B surface antigen; HBsAg, hepatitis B surface antigen; HBV, hepatitis B virus; NA, not applicable.

### Safety

**Figure 2:**
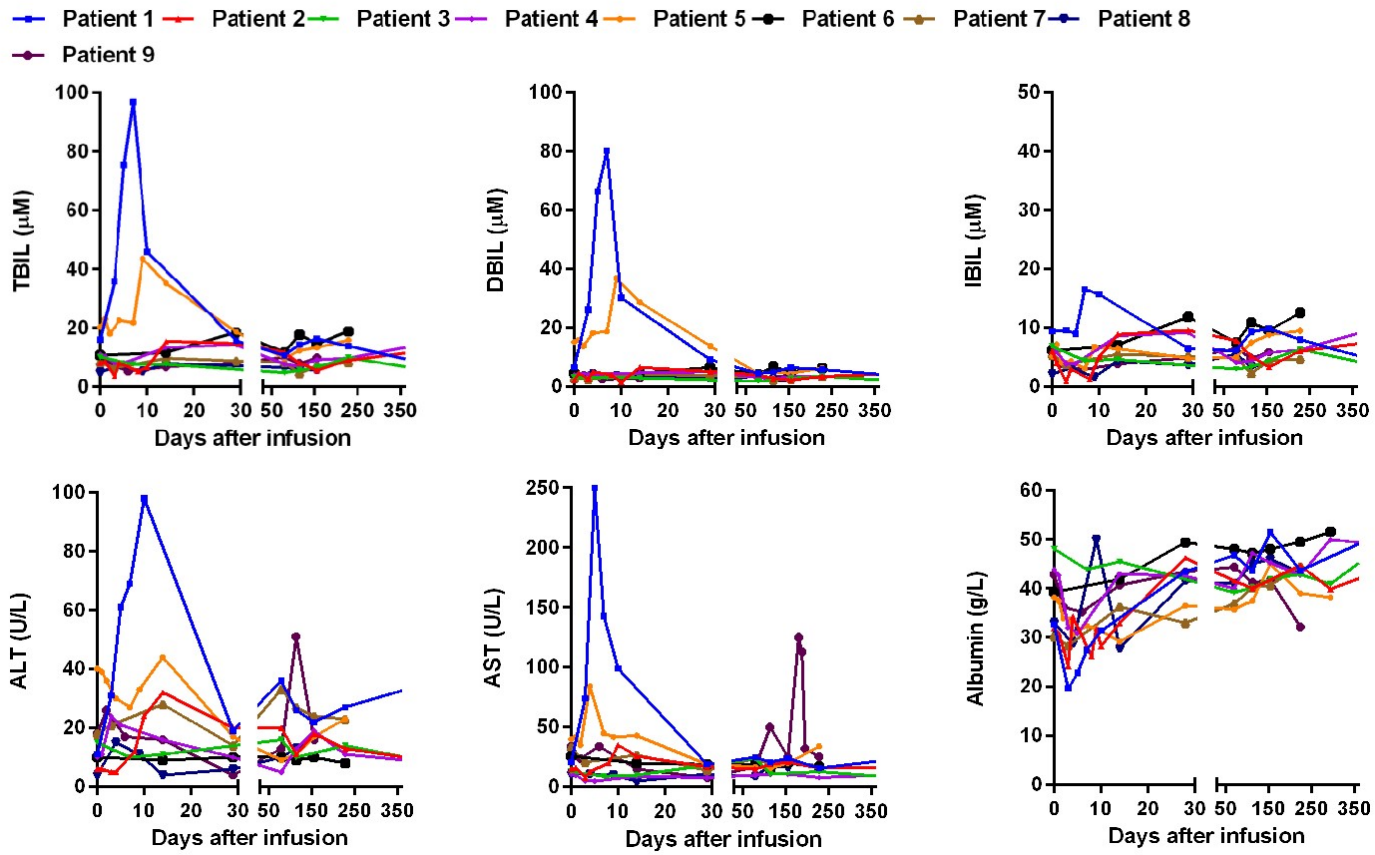
Hepatic function of BCMA CAR-T in MM with chronic HBV infection. Expression of TBIL (normal range, 0 to 21 µM), DBIL (normal range, 0 to 5 µM), IBIL (normal range, 0 to 15 µM), ALT (normal range, 5 to 40 U/L), AST(normal range, 8 to 40 U/L) and albumin (normal range, 34 to 48 g/L) after CAR-T cells infusion. ALT, alanine transaminase; AST, aspartate transaminase; CAR, chimeric antigen receptor; DBIL, direct bilirubin; IBIL, indirect bilirubin; TBIL, total bilirubin.

Prophylaxis with antiviral drugs was recommended for all nine patients with MM. However, patient 6 (HBsAg positive) had been treated with entecavir/lamivudine; patients 2 and 3, who had a resolved HBV infection, had antiviral drug prophylaxis; other patients did not receive antiviral prophylaxis for various reasons after the CAR infusion. The bilirubin, transaminase, and albumin levels were determined after the infusion (figure 2). The median peak total bilirubin (TBIL) level was 14.3 µM (range, 6.5–96.8 µM; normal range, 0–21 µM), the median peak direct bilirubin (DBIL) level was 5.1 µM (range, 4.1–80.2 µM; normal range, 0–5 µM), and the median peak indirect bilirubin (IBIL) level was 9.2 µM (range, 5.5–16.6 µM; normal range, 0–15 µM). The median peak alanine transaminase (ALT) level was 32 U/L (range, 10–98 U/L; normal range, 5–40 U/L), and the median peak aspartate transaminase (AST) level was 27 U/L (range, 11–250 U/L; normal range, 8–40 U/L). The median low albumin level was 30.7 g/L (range, 19.6–40.2 g/L; normal range, 34–48 g/L). Among which, patient 1 had elevated TBIL, DBIL, IBIL, ALT, and AST on days 3–10, up to grade 4 adverse events; patient 5 had elevated TBIL, DBIL, ALT and AST on days 3–10, up to grade 3 adverse events (online supplementary table 2); patients 1, 2, 4, 7, and 8 had reduced albumin on days 3–10. The abnormalities in these patients may be related to CRS or CT. During later follow-up, patient 9 had elevated ALT, AST on days 124–131 (up to grade 1 adverse events), and had elevated AST on days 180–190 (up to grade 2 adverse events, online supplementary figure 1); the other patients had no obvious abnormalities in bilirubin, transaminase, or albumin.

HBV serologies and HBV DNA copy numbers were monitored for all patients after the CAR-T cells infusion. For patient 6 (HBsAg positive), although the HBV DNA replication was below the lower limit of detection after the infusion, the lack of dynamic detection for HBV DNA levels during and after CAR-T cell therapy may be a limitation to this study. Of the patients who had resolved HBV infection, patient 9 was HBsAb and HBcAb positive before the CAR-T cells infusion, but her HBsAb became negative on day 28 after CAR-T cells infusion, and HBsAg appeared positive on day 180 after CAR-T cells infusion, indicating that HBV reactivation occurred in this patient. She was given entecavir (1 mg/day) at this time. Two months later antiviral therapy, her HBsAg became negative, HBsAb appeared positive. The HBV DNA level was checked two times before and after the HBsAg changed positive again, and found it was all below the detectable limit (online supplementary table 3). The other patients did not exhibit positivity for HBsAg or HBV DNA replication (table 2). In addition, for the patient 6 (HBsAg positive), the aspartate aminotransferase-to-platelet ratio index （APRI） score was <0.5 before and after BCMA CAR-T cell therapy, the FIB-4 score was 1.85 before treatment and 1.10–2.02 after CAR-T cell therapy (online supplementary table 4), which showed that CAR-T therapy did not affect the degree of liver fibrosis.

**Figure 3:**
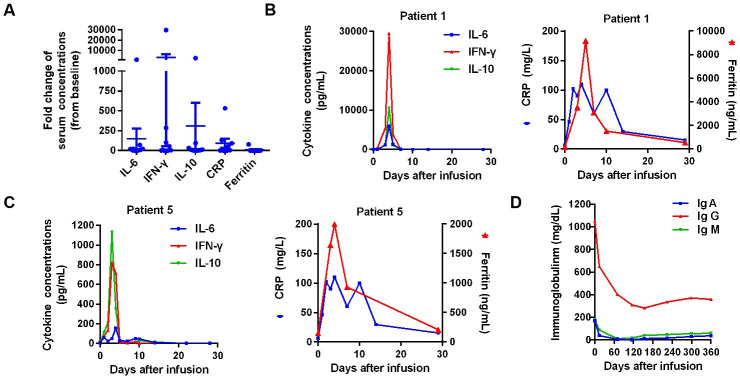
Adverse events of BCMA CAR-T cell therapy in R/R MM. (A) Fold change (peak concentration compared with baseline) of serum cytokines (IL-6, IL-10, and interferon (IFN)-γ), ferritin, and CRP after CAR-T cells infusion in patients with MM. (B, C) Changes in the levels of cytokines, serum ferritin, and serum CRP in representative cases (patient 1 and patient 5) after CAR-T cells infusion. (D) Changes in serum immunoglobulin (Ig)A (normal range, 90 to 450 mg/dL), IgG (normal range, 800 to 1800 mg/dL), and IgM (normal range, 60 to 280 mg/dL) levels after CAR-T cell therapy. BCMA, B cell maturation antigen; CAR, chimeric antigen receptor; CRP, C-reactive protein; MM, multiple myeloma; R/R, refractory/relapsed.

Eight patients had adverse events (table 3). The most common events of grade 3 or higher were hematologic toxic effects, including neutropenia, leukopenia, anemia, and thrombopenia; the adverse events may be due to toxicity caused by lymphodepleting CT. Seven (77.78%) patients had CRS. A higher incidence of CRS was also associated with high cytokine secretion and increased levels of serum CRP and serum ferritin (figure 3A). CRS occurred early after the CAR-T cells infusion, with a median time of onset of 1 day (range, 1–3 days) and a median duration of 4 days (range, 1–10 days). Figure 3B, C shows representative results for patient 1 and patient 5, separately. Other symptoms related to CRS are shown in table 3. None of the patients had CAR-related encephalopathy syndrome.

**Table 3:** Adverse effects of CAR-T and their management

Patient	Cytopenia (AE grading)	CRS (AE grading)	CRES	CRS grading	Use of IL-6R inhibitor	Use of glucocorticoid
1	PLT↓ (3)	Fever (3), Diarrhea (2), Creatinine ↑ (1), Abnormal coagulation (1)	No	2	Yes	Yes
2	WBC↓ (3)	Fever (2), Hypoxia (3), Chill (1), Heart failure (3), hypotension (3)	No	3	No	Yes
3	No	No	No	0	No	No
4	WBC↓ (2), NEU↓ (3), PLT↓ (3), LYM ↓ (2)	Fever (2), Hypoglobulinemia (1)	No	1	No	No
5	No	Fever (1), Bilirubin ↑ (1), D-Dimer (1), LDH ↑ (1),α-HBDH ↑ (1)	No	1	No	No
6	No	No	No	0	No	No
7	WBC↓ (4), NEU↓ (4), PLT↓ (4), LYM↓ (3), MON↓ (2)	Fever (2)	No	1	No	No
8	WBC↓ (4), NEU↓ (4), PLT↓ (4), LYM ↓ (4)	Fever (1), NT-BNP (1)	No	2	No	No
9	LYM ↓ (4)	Fever (2)	No	1	No	No

↓ indicates decrease; ↑ indicates increase.

The grading of AE was according to the CTCAE V.4.03.

AEs, adverse events; CRES, CAR-related encephalopathy syndrome; CRS, cytokine release syndrome; α-HBDH, α hydroxybutyrate dehydrogenase; LDH, lactate dehydrogenase; LYM, lymphocyte; NEU, neutrophil count; NT-BNP, N-terminal B-type natriuretic peptide; PLT, platelet count; WBC, white blood cell.

All patients receiving CAR-T cell therapy had low levels of IgG, IgA, and IgM at 1 month, and these low levels lasted for at least 3 months to more than 1 year (figure 3D and online supplementary tables 5–7). All patients received Ig replacement infusion.

### Efficacy

**Figure 4:**
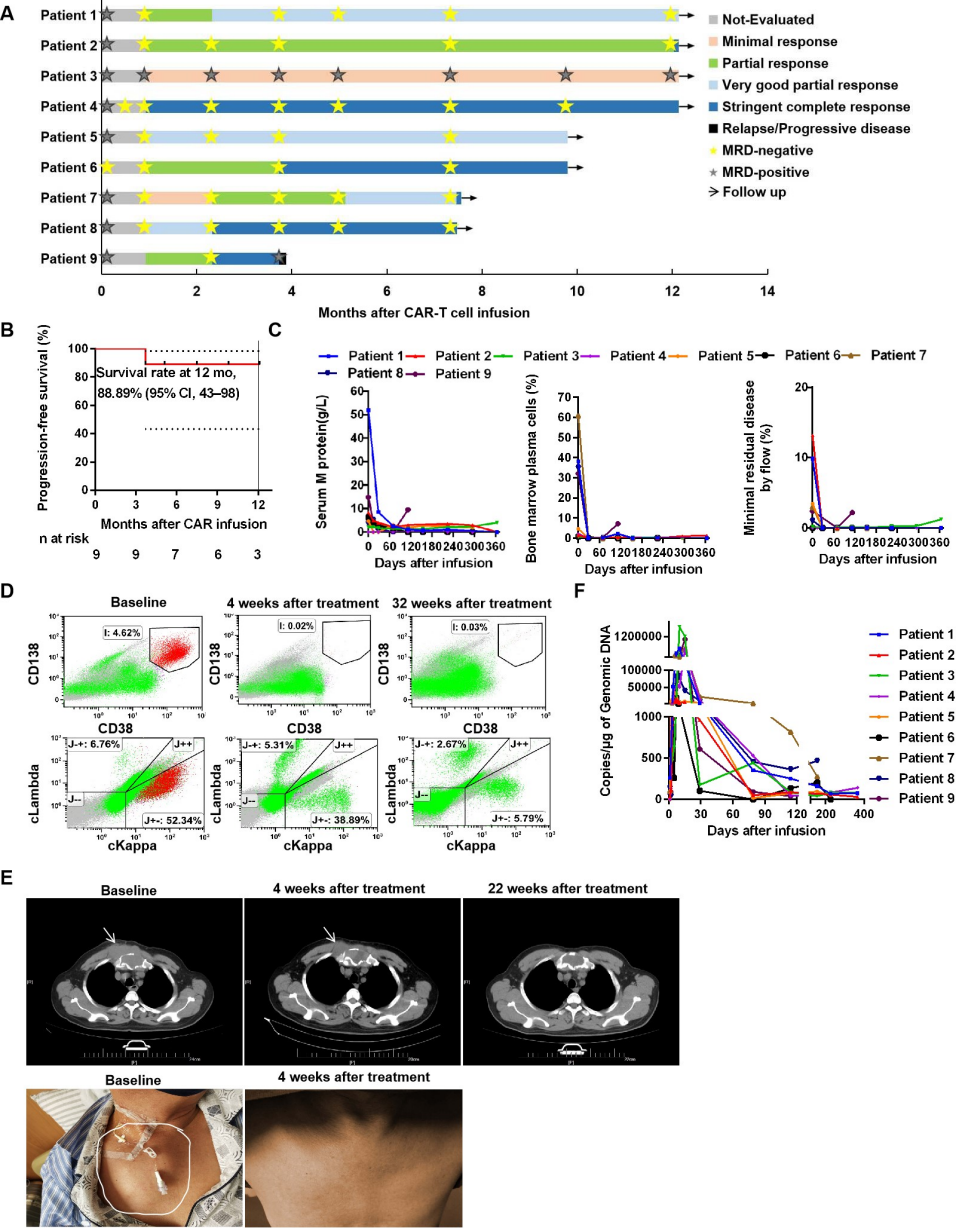
Clinical activity after BCMA CAR-T cells infusion in patients. (A) Best responses among the nine patients. All responses were confirmed and assessed according to the International Myeloma Working Group uniform response criteria for MM. MRD denotes minimal residual disease based on flow cytometry analysis. (B) Prognosis of patients with MM after the BCMA CAR-T cells infusion. The PFS was calculated by the Kaplan-Meier method. (C) Changes in plasma M-protein concentrations (left), plasma cells in the bone marrow by bone marrow cytological examination (middle), and MRD by flow cytometry (right) after BCMA CAR-T cell therapy. (D) Flow cytometry analysis of bone marrow plasma cells in a representative case (patient 5) after BCMA CAR-T cells treatment. No abnormal plasma cells and light chain-related tumor cells were found on reexamination. (E) Response of extramedullary infiltration lesions in a representative case (patient 6) after CAR-T cells infusion. The mass in the chest was reduced in size at 4 weeks and then disappeared at 22 weeks, as demonstrated by CT and surface observation. white arrows indicate sites of tumor lesions. (F) Gene-modified T cells in peripheral blood were assessed by quantitative real-time PCR. The horizontal line at 5 copies/µg DNA represents the lower limit of quantification of this assay. Data at the first time point were obtained before infusion of BCMA CAR-T cells. BCMA, B cell maturation antigen; CAR, chimeric antigen receptor; PFS, progression-free survival.

At 1 month after the CAR-T cell therapy, all patients (100%) achieved an objective response (figure 4A). During the follow-up period from 3.7 to 12.1 months (median, 9.8 months) for nine patients, as of November 22, 2019, six (66.67%) patients achieved stringent CR (sCR), two (22.22%) patients exhibited very good partial response (PR), and one (11.11%) patient exhibited minimal response (figure 4A). Notably, although CR was achieved early in some cases, efficacy improved over time in other patients. Relapse occurred in one patient after sCR. Kaplan-Meier curve analysis showed a PFS rate of 88.89% (95% CI, 43%–98%) at 12 months (figure 4B); the median PFS was not determined. Because of the small sample size in our study, subgroups could not be analyzed according to tumor BCMA levels, baseline serum results, previous treatment exposure, high-risk cytogenetic profiles, CRS, and CAR-T cells expansion in vivo.

Responses occurred early, and the median time to the first PR or better was 4 weeks (range, 4–10 weeks). Serum M protein levels declined slowly, leading to a gradual improvement in the response over time in some patients. Compared with the baseline levels, the plasma cells were completely cleared within 4 weeks in most patients, except for patients 7 and 9 (figure 4C); in addition, nearly complete decrease (>90%) from the baseline was observed in tumor-associated serum FLC within 4 weeks. Eight patients (excluding patient 3) were MRD negative, with an estimated 10^5^ nucleated cells at 4 weeks. During follow-up, patient 9 became MRD positive at week 16, whereas all other patients were MRD negative at the post-treatment efficacy evaluation (for which the longest duration was more than 12 months; see figure 4A, C and D for representative results for patient 5). Tumor responses in sites of extramedullary disease were also observed by 4 weeks, for example, for patient 6 (figure 4E).

The levels of CAR-T cells were easily detected by the CAR DNA copy number, and the results showed high in vivo proliferation (figure 4F). The number of CAR-T cells typically began to increase on the second day and peaked at 5–20 days. The median peak CAR DNA copy number was 512 537.14 copies/µg genomic DNA (range: 6846.63–1,716,880.39 copies/µg genomic DNA). The peak values were not dependent on the initial dose of CAR-T administered (table 1 and figure 4F). In addition, the CAR-T cells expansion in patients 1 and 2 did not appear to be negatively affected by tocilizumab or glucocorticoid use.

## Discussion

This is the first systematic analysis of the safety and efficacy of BCMA CAR-T cell therapy in patients with MM and concomitant HBV infection. Our findings showed that BCMA CAR-T cells could be safely used to treat patients with HBV-infected MM, if antiviral drugs were administered during the therapy; the curative effects of CAR-T cells did not seem altered by the infection of HBV.

MM is a hematological malignancy with no cure. Despite advancements in treatment strategies, including IMiD, PI, and monoclonal antibodies,^24–26^ almost all patients with a high-risk cytogenetic profile or treatment-refractory disease eventually relapse.^27–29^ Recently, BCMA CAR-T cells have been shown to have promise in the treatment of MM.^19 20 30^ In addition, some reports have shown that the prevalence of HBV infection is high in patients with MM.^31 32^ However, to avoid viral reactivation or fulminant hepatitis, clinical trials of CAR-T therapies have generally excluded patients with aggressive MM who are coinfected with HBV. Accordingly, it was unclear whether CAR-T therapy could be effectively applied to HBV-infected patients with MM.

Patients who were HBsAg positive with malignant tumors may experience HBV reactivation during anticancer treatment, such as CT.^11^ Delayed HBV reactivation has occurred months after anticancer therapy by rituximab because of immunosuppression with progressive B cell depletion.^33–35^ CAR-T cell therapy may be more likely to cause HBV reactivation because it is more complex than common methods of MM therapy. High-intensity CT with fludarabine and cyclophosphamide is required before CAR-T cells infusion, and serious CRS may occur after the infusion; any of these may cause HBV reactivation. However, Strati *et al* were the first to report the safe treatment of a patient who was HBsAg positive with diffuse large B cell lymphoma (DLBCL) with CD19 CAR-T cells.^36^ This patient remained on tenofovir prescribed at the same dose, before and after the CAR-T cells infusion. The patient had grade 3–4 CRS and achieved CR, with a PFS time of 8 months after therapy, until the follow-up cut-off, and with no reactivation of HBV or significant increase of ALT and bilirubin. Similar to the report by Strati *et al*,^36^ we also treated an patient (patient 6) with HBsAg positive MM with BCMA CAR-T cells under the protection of entecavir. This patient achieved PR and remained at the sCR state after the 9.8 month observation. Most importantly, this patient did not experience HBV reactivation. These results indicate that CAR-T cells can safely and effectively be used to treat patients who were HBV-infected under the protection of anti-HBV drugs, even patients who were HBsAg positive. Wei J *et al*^37^ reported that a patient who had HBsAg-positive DLBCL died of HBV reactivation during CAR-T cell therapy because the patient discontinued antiviral drugs, suggesting the importance of concomitant antiviral and CAR-T cell therapy in patients with cancer who were HBV-infected.

Although HBV reactivation has been reported in patients with resolved HBV infection during the application of new anticancer drugs,^4 38 39^ CAR-T cell therapy in patients with MM who have resolved HBV may be safer than that in patients who were HBsAg positive. In our present study, of the eight patients with MM who have resolved HBV infection, two patients administered prophylactic anti-HBV drugs did not exhibit HBV reactivation; of the six patients who did not use prophylactic antiviral drugs, five did not exhibit HBV reactivation, only one showed recurrence of HBsAg without detection of HBV DNA or damage to liver function. However, among the six patients who did not use prophylactic antiviral drugs, two patients had elevated liver function within the early 2 weeks. Although the abnormal liver function might be associated with CRS or CT, hepatitis B serology and HBV DNA levels should be checked at this time to distinguish if this was caused by HBV reactivation. The lack of hepatitis B serology and HBV DNA in this study is a limitation for the diagnoses of the two patients. These above results suggest that CAR-T cell therapy is safe for patients with MM who have resolved HBV. While prophylaxis is recommended for all patients, for those who cannot or do not take prophylaxis, CAR-T cell therapy may still be feasible with close laboratory monitoring of HBV serologies and HBV DNA, and prompt initiation of antiviral therapy if turning positive. Strati *et al*^36^ reported that a patient with DLBCL who had resolved HBV exhibited HBV DNA reactivation after 3 months because of self-discontinued antiviral prophylaxis 13 months after infusion; this indicates that anti-HBV therapy should be administered until the patient serum Ig has recovered. In addition, in the present study, of the six patients who did not use prophylactic antiviral drugs, only one had evidence for reactivation; this may be associated with patients receiving intravenous immunoglobulin replacement, which contributed to the low re-activation rate.

Another risk of CAR-T cell therapy is that the HBV infection aggravates CRS. Fortunately, of the nine patients who were HBV-infected in our study, no patient died of CRS after CAR-T cell therapy. Six patients experienced grade 1–2 CRS, and one patient experienced grade 3 CRS, all of whom were controlled by symptomatic support and IL-6 therapy. These results indicated that the HBV infection did not aggravate CRS; however, further studies are needed to clarify the risk of CRS in the presence of chronic HBV infection. Importantly, the HBV infection did not seem to affect the effectiveness of the CAR-T cell therapy; of the nine patients with MM treated, the best ORR was 100%, and the PFS rate at 12 months was 88.89%, which were comparable with the curative effects of other clinical trials.^19 20^

In conclusion, for patients with MM with resolved HBV infection, BCMA CAR-T cell therapy can be administered safely and the clinical efficacy dose not seem to be affected. However, strictly monitor the HBV infection and concurrently administer antiviral therapy are recommended to prevent the reactivation of HBV. Because of the small number of patients and the retrospective nature of this study, additional clinical research is needed to clarify the safety and efficacy of this approach.
