# Supplementary material for: Safety and efficacy of CAR-T cell targeting BCMA in patients with multiple myeloma coinfected with chronic hepatitis B virus
Source: J Immunother Cancer. 2020 Aug 13;8(2):e000927. doi: 10.1136/jitc-2020-000927 (PMC7430488; doi:10.1136/jitc-2020-000927)
Supplement: Supplementary data [file jitc-2020-000927supp001.pdf]

Supplementary Materials

BCMA CAR-T cell therapy in patients with multiple myeloma and chronic hepatitis B virus infection

Lu Han, Jian Zhou, Ke-Shu Zhou, Xing-Hu Zhu, Ling-Di Zhao, Bai-Jun Fang, Qing-Song Yin, Xu-Dong Wei, Hu Zhou, Lin-Lin Li, Beng-Ling Xu, Ji-Shuai Zhang, Yong-Ping Song*, Quan-Li Gao*

Inclusion Criteria

Patients enrolled in the enrollment accord with the following conditions in this study.

The criteria set include the diagnosis of multiple myeloma, the lack of effective treatment options (such as autologous or allogenic stem cell transplantation) and the limited overall survival time after treatment with existing treatments, as follows:

1. Age: 18~75 years old, expected survival period is greater than 3 months;
2. Confirmed as active MM according to IMWG, and confirmed as BCMA positive MM by immunohistochemistry or flow cytometry;
3. According to any of the following criteria, there were measurable indexes: serum monoclonal protein (M protein) in serum ≥ 1.0 g/dL or urine M protein level ≥ 200 mg/24 hours; serum free light chains (FLC) (involved FLC concentration of ≥ 10 mg/dL with abnormal ratio); bone marrow plasma cells ≥ 10%;
4. At least three previous lines of therapy, each line of treatment has at least 1 complete treatment cycle, except the best response to the treatment regimen was recorded as progressive diseases (according to the IMWG criteria);
5. Received an immunomodulatory drug (IMiD) and a proteasome inhibitor (PI), or disease refractory to both drug classes; and adequate organ function;
6. The patients enrolled in this subjects needed to be progressing after most recent therapy and had measurable disease simultaneously. Disease progression must be proved by objective examination data.
7. ECOG score 0-2;

Han L, et al. J Immunother Cancer 2020; 8:e000927. doi: 10.1136/jitc-2020-000927

8. The clinical laboratory values during screening period met the following criteria: hemoglobin ≥ 60 g/L (no red blood cell was infused in the first 7 days of laboratory examination; recombinant human erythropoietin was permitted to use), platelet ≥ 30 × 10^9/L (blood transfusion must not be supported by infusion fraction within 7 days before laboratory examination); the absolute value of neutrophils ≥ 0.75 × 10^9/L (growth factor support is allowed to use, but no support treatment must be received within 7 days of laboratory examination); AST and ALT ≤ 2.5 × normal upper limit (ULN); total bilirubin ≤ 3.0 × ULN (direct bilirubin ≤ 1.5× ULN is required for subjects with preemptive hyperglycemia, such as Gilbert syndrome); renal function Cr ≤ 1.25 ULN; corrected serum calcium ≤ 12.5 mg/dL or free ion calcium ≤ 6.5 mg/dL;

9. In addition to hair loss or peripheral neuropathy, the toxicity of prior anti-tumor treatment must be returned to a baseline level or ≤ level 1;

10. There are no other serious diseases (such as autoimmune diseases, immunodeficiencies, organ transplants) that conflict with this program;

11. No history of other malignant tumors;

12. Within 7 days before the trial, women in childbearing age should do a blood pregnancy test and the result should be negative; and women in childbearing age must adopt appropriate contraceptive measures during the trial period and within 3 months after the trial;

13. The patient agrees to participate in the clinical study and sign the informed consent form.

Exclusion Criteria

Any exclusion criteria are not eligible for inclusion:

1. Pregnant or lactating women (for women in childbearing age, a pregnancy check is necessary);

2. Patients who suffer from severe infectious diseases or viral diseases (HIV positive, syphilis, etc.);

3. Active hepatitis B or C;

4. Patients who have systematically used large amounts of glucocorticoids with 1 week before enrollment;

5. Patients who suffer from severe heart, liver, kidney dysfunction, diabetes or other diseases;

6. Patients who have been in other clinical studies in the past 3 months or who have been treated with other gene products;

Preparation and Phenotype of CAR-T Cells

The BCMA CAR-T cells were derived from autologous peripheral blood mononuclear cells, and $CD3^+$ T cells were isolated by positive magnetic selection (Miltenyi, Bergisch Gladbach, Germany). Cell purity was detected using a FACSCanto II system (BD Biosciences, San Jose, CA, USA) and staining with anti-CD3-APC (Biolegend, San Diego, CA, USA). $CD3^+$ T cells were stimulated with Retronectin (Takara Bio, Otsu, Japan) and the anti-CD3 mAb (Takara Bio). The cells were transduced 1 day after activation with a lentiviral vector, containing the anti-BCMA CAR (offered by Shenzhen Pregene Biopharma Company, Ltd.), and expanded over a period of 12 days. All cells were expanded and analyzed by flow cytometry, until day 12. Cells were harvested, washed in cold phosphate-buffered saline (PBS), and resuspended at 1×10^6 in 100 μL of cold PBS comprising 5% serum. Cells were stained with Biotinylated Human BCMA, Fc Tag (BC7-H82F0, ACRO), anti-CD4-PE-Cy7, anti-CD8-PerCP-Cy5.5, anti-CD45RA-APC-Cy7, anti-CCR7-APC (all purchased from Biolegend) for 20 min on ice. FACS analysis was performed using a FACSCanto II system and CellQuest software (BD Biosciences).

Treatment Administration

Patients received lymphodepletion with fludarabine (25–30 mg/m^2 on days −5 to −3) and cyclophosphamide (600–800 mg/m^2 on days −5 to −4) followed by infusion of CAR-T on day 0. On day −1, weeks 4, 10, 16, 22 and every 10 weeks thereafter, efficacy were performed. The dose was 5×10^6 CAR-positive T cells/kg. The positive percentage of BCMA CAR-T cells was (32.33 ± 14.77)%. The percentage of $CD4^+$ cells and $CD8^+$ cells were (32.63 ± 13.86)%, (65.33 ± 12.11)% respectively. In addition, effector memory ($CD45RA^-$ $CCR7^-$) and differentiated effector memory ($CD45RA^+$ $CCR7^-$) cells account for the main proportion in $CD4^+$ and $CD8^+$ cell

subpopulation (Supplementary Table 1).

Cytokine Secretion

Cytokines, including IL-6, IL-10 and IFN-γ, using ELISA kits (R&D Systems, Minneapolis, MN, USA) according to manufacturer protocol. All samples were analyzed in triplicate and compared against multiple internal standards using standard curves. Data was acquired, and analysis was performed using MasterPlex ReaderFit (Hitachi Solutions America, Ltd., Irvine, CA, USA).

Assessment of CAR-T Cells by Quantitative Real-Time PCR

CAR DNA copy numbers were determined for evaluating CAR-T cell expansion and persistence. Genomic DNA was extracted using a QIAamp DNA Blood Mini Kit (Qiagen, Hilden, Germany) from fresh peripheral blood samples after CAR-T cell infusion. CAR DNA copy numbers were assessed by quantitative real-time PCR.

Han L, *et al. J Immunother Cancer* 2020; 8:e000927. doi: 10.1136/jitc-2020-000927

Results

Supplemental Figure 1. The change of bilirubin (left panel), transaminase and albumin (right panel) levels with patient 9 who has seroreversion of the HBsAg after CAR-T cells infusion. TBIL, total bilirubin (normal range, 0–21 µM); DBIL, direct bilirubin (normal range, 0–5 µM); IBIL, indirect bilirubin (normal range, 0–15 µM); ALT, alanine transferase (normal range, 5–40 U/L); AST, aspartate transaminase (normal range, 8–40 U/L); ALB, albumin (normal range, 34–48 g/L).

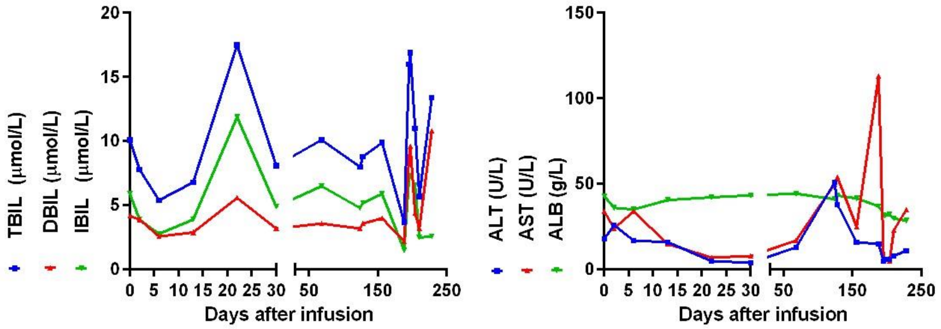

BMJ Publishing Group Limited (BMJ) disclaims all liability and responsibility arising from any reliance placed on this supplemental material which has been supplied by the author(s)

Supplementary Table 1. Information of CAR-T cells in product, conditioning therapy and method of infusion.

Patient	Fraction of CAR$^+$ T cells (%)	CD4$^+$ cells (%)	CD4$^+$ cells				CD8$^+$ cells (%)	CD8$^+$ cells				Conditioning therapy*	Method of infusion
			CD45RA$^+$ CCR7$^+$ (%)	CD45RA$^-$ CCR7$^+$ (%)	CD45RA$^-$ CCR7$^-$ (%)	CD45RA$^+$ CCR7$^-$ (%)		CD45RA$^+$ CCR7$^+$ (%)	CD45RA$^-$ CCR7$^+$ (%)	CD45RA$^-$ CCR7$^-$ (%)	CD45RA$^+$ CCR7$^-$ (%)		
1	40	34.9	5	5.2	84.5	5.4	61.9	6.6	2.6	68.6	22.2	FC	one-infusion
2	55	55.8	2.2	6.4	69.3	22	52.4	40.9	19.6	15.1	24.3	FC	one-infusion
3	23	15.3	0.7	1.1	96.7	2.5	81.1	0.8	1	93.5	4.6	FC	one-infusion
4	40	24.4	0.8	0.3	75.8	23	71.2	3.4	1.7	44.4	50.5	FC	one-infusion
5	13	15.6	0.9	0.6	51.2	47.3	83.3	3.9	0.2	6	89.9	FC	one-infusion
6	5	22.6	0.2	0.1	42.8	56.9	76.8	4.3	0.2	7.2	88.2	FC	one-infusion
7	40	44.2	0.9	0.5	48.2	50.4	54	3.9	0.1	15	80.9	FC	one-infusion
8	35	44.5	0.3	1.1	87.8	14.7	52.9	2.8	2.6	52.8	41.8	FC	one-infusion
9	40	45.4	14	26.1	42	17.9	54.4	5.5	3.2	26.7	64.6	FC	one-infusion

Note: * F, Fludarabine 25—30 mg/m^2 intravenously daily for 3 days; C, Cyclophosphamide 600—800 mg/m^2 intravenously daily for 2 days.

Supplementary Table 2. An elevation in bilirubin and transaminase levels for patients 1 and 5 after CAR-T cells infusion.

Patient	TBIL	DBIL	IBIL	ALT	AST	AE grading
1	4.6 × ULN	16 × ULN	1.1 × ULN	2.5 × ULN	6.3 × ULN	4
9	2.1 × ULN	7.4 × ULN	Normal	1.1 × ULN	1.1 × ULN	3

TBIL, total bilirubin; DBIL, direct bilirubin; IBIL, indirect bilirubin; ALT, alanine transferase; AST, aspartate transaminase; ULN, the normal upper limit. AE, adverse events (the grading of AE was according to the CTCAE 4.03).

Supplementary Table 3. The change of HBV serology with patient 9 who has seroreversion of the HBsAg after CAR-T cells infusion.

Day	HBsAg	HBsAb	HBeAg	HBeAb	HBcAb	HBV DNA	Antivira therapy
Day 0	Negative	Positive	Negative	Negative	Positive	<100 IU/mL	No
Day 28	Negative	Negative	Negative	Negative	Positive	--	No
Day 180	Positive	Negative	Negative	Negative	Positive	<100 IU/mL	Entecavir
Day 256	Negative	Positive	Negative	Negative	Positive	--	Entecavir

--, No assessment.

Supplementary Table 4. The fibrosis stage of patient 6 ($HBsAg^+$).

Parameter	Day 0	Day 17	Day 28	Day 70	Day 112	Day 154	Day 224
APRI score	0.25	0.19	0.27	0.23	0.14	0.24	0.20
FIB-4 score	1.85	1.46	2.02	1.70	1.10	1.80	1.70

APRI score, Aspartate aminotransferase-to-Platelet Ratio Index (With an APRI threshold of 0.5, 1.0 and 1.5, the sensitivity & specificity values were 70.0% & 60.0%, 50.0% & 83.0% and 36.9% & 92.5% for significant fibrosis, advanced fibrosis and cirrhosis, respectively).[1-3] FIB-4 score, Fibrosis-4 Score (FIB-4 index <1.45 had a negative predictive value of 94.7% to exclude severe fibrosis with a sensitivity of 74.3%; FIB-4 index higher than 3.25 had a positive predictive value to confirm the existence of a significant fibrosis (F3-F4) of 82.1% with a specificity of 98.2%).[4, 5]

Supplementary Table 5. The change of immunoglobulin (Ig) A in relapsed/refractory multiple myeloma after CAR-T infusion.

Patient	Day 0	Day 14	Day 28	Day 70	Day 112	Day 154	Day 224	Day 294	Day 364
1	29.3	26.3	12.9	0.3	14.5	38.8	15	--	32.8
2	84	30.9	7.4	13.9	46.3	--	58.1	42.4	63.4
3	50.9	21.3	13.9	24.2	7.6	12.8	19.4	24.9	21.3
4	171.2	39.5	--	6.8	2.7	12.2	17.7	30.7	38.3
5	30.9	13.1	6.4	5	16.4	31.9	35.7	49.8	--
6	64.4	--	5.4	9.4	15.2	21.3	29.3	31.2	--
7	60.7	31.6	15.5	2.5	0.2	6.5	4.9	--	--
8	1874.9	1058.6	275.9	1.5	11.4	22.5	99.8	--	--
9	879.3	345.8	44.9	18	1911.9	--	--	--	--

--, No assessment. Normal range: 90–450 mg/dL.

Supplementary Table 6. The change of immunoglobulin (Ig) G in relapsed/refractory multiple myeloma after CAR-T infusion.

Patient	Day 0	Day 14	Day 28	Day 70	Day 112	Day 154	Day 224	Day 294	Day 364
1	8177	2936	1377	423	268	369	370	--	524
2	2065	1295	953	538	998	--	1248	1494	1257
3	579	546	--	318	--	386	636	587	--
4	1052	650	--	404	311	283	336	372	360
5	567	345	289	505	613	902	1021	1059	--
6	1574	--	614	445	741	1169	1096	1195	--
7	1451	1186	778	437	310	156	100	--	--
8	309	160	3.3	165	522	433	589	--	--
9	577	442	316	372	247	--	--	--	--

--, No assessment. Normal range: 800–1800 mg/dL.

Supplementary Table 7. The change of immunoglobulin (Ig) M in relapsed/refractory multiple myeloma after CAR-T infusion.

Patient	Day 0	Day 14	Day 28	Day 70	Day 112	Day 154	Day 224	Day 294	Day 364
1	19.2	19.8	13.7	33.2	30.8	62.5	49.6	--	59.3
2	19.3	12.2	7.3	16.7	64.4	--	184.2	70.8	71.4
3	22.1	8.2	--	11	--	17.9	14.7	24.2	--
4	179.1	88.3	--	12	17.7	40	48	57	59.6
5	24.7	12	16	14.5	40.3	49.3	61.8	70.2	--
6	81.8	--	19.9	26.2	49.1	46.1	49.6	71.9	--
7	6.2	6.6	12.6	3.5	0.9	29.6	77.4	--	--
8	23.9	17.7	11.33	7.1	39	35.8	106.6	--	--
9	38.8	25.3	11.4	34.3	36.4	--	--	--	--

--, No assessment. Normal range: 60–280 mg/dL.

References

1. WHO. Guidelines for the prevention, care and treatment of persons with chronic hepatitis B infection. 2015-3-12. http://www.who.int/hiv/pub/hepatitis/hepatitis-b-guidelines/en/.
2. Lin ZH, Xin YN, Dong QJ, et al. Performance of the aspartate aminotransferase-to-platelet ratio index for the staging of hepatitis C-related fibrosis: an updated meta-analysis. Hepatology. 2011. 53(3): 726-36.
3. Xiao G,Yang J, Yan L. Comparison of diagnostic accuracy of aspartate aminotransferase to platelet ratio index and fibrosis-4 index for detecting liver fibrosis in adult patients with chronic hepatitis B virus infection: a systemic review and meta-analysis [J]. Hepatology,2015,61(1):292-302.
4. Vallet-Pichard A, Mallet V, Nalpas B, et al. FIB-4: an inexpensive and accurate marker of fibrosis in HCV infection. comparison with liver biopsy and fibrotest. Hepatology. 2007. 46(1): 32-6.
5. Li Y, Chen Y, Zhao Y. The diagnostic value of the FIB-4 index for staging hepatitis B-related fibrosis: a meta-analysis. PLoS One. 2014. 9(8): e105728.
